# Influenza A(H1N1)pdm09 Virus among Healthy Show Pigs, United States

**DOI:** 10.3201/eid1809.120431

**Published:** 2012-09

**Authors:** Gregory C. Gray, Jeffrey B. Bender, Carolyn B. Bridges, Russell F. Daly, Whitney S. Krueger, Michael J. Male, Gary L. Heil, John A. Friary, Robin B. Derby, Nancy J. Cox

**Affiliations:** University of Florida, Gainesville, Florida, USA (G.C. Gray, W.S. Krueger, G.L. Heil, J.A. Friary, R.B. Derby);; University of Minnesota, St. Paul, Minnesota, USA (J.B. Bender);; Centers for Disease Control and Prevention, Atlanta, Georgia, USA (C.B. Bridges, N.J. Cox);; South Dakota State University, Brookings, South Dakota, USA (R.F. Daly);; and University of Iowa, Iowa City, Iowa, USA (M.J. Male)

**Keywords:** epidemiology, influenza, influenza A virus, porcine, occupational exposure, pigs, South Dakota, Minnesota, viruses, zoonoses, United States, pandemic influenza virus

## Abstract

Within 5 months after the earliest detection of human influenza A(H1N1)pdm09 virus, we found molecular and culture evidence of the virus in healthy US show pigs. The mixing of humans and pigs at swine shows possibly could further the geographic and cross-species spread of influenza A viruses.

## Abstract 2

Because animals can transmit some diseases to people, it is wise to be cautious around animals that carry these diseases. But how do you know which animals are carrying disease? Sometimes they appear perfectly healthy. A study of 57 apparently healthy show pigs at a 2009 US state fair found that almost 20% were carrying influenza virus and at least 4 were carrying the 2009 pandemic virus. Of concern is the possibility that different types of influenza virus—pandemic, swine, avian—could combine in pigs and emerge as new viruses that then spread to humans. Swine workers, veterinarians, and other persons with pig contact may be at high risk for infection with pig influenza and should receive seasonal influenza vaccines, use personal protective equipment when working with healthy pigs, and limit their contact with sick pigs. Regular monitoring of influenza virus among pigs and testing of sick persons who have been exposed to pigs are needed.

Cross-species infections with influenza A viruses readily occur between humans and pigs. Pigs often have been infected by human epidemic viruses (*1*), and swine workers and their family members are at increased risk for swine influenza virus (SIV) infection (*2*–*4*). We studied swine shows as a setting for influenza A virus transmission (*5*).

## The Study

After acquiring informed consent, we recruited persons >7 years of age showing pigs at 3 state fairs in Minnesota (2008, 2009) and South Dakota (2009). Exhibitors were eligible for the study if they reported working with pigs at least 1 cumulative hour per week and had no current immunocompromising condition. Enrolled participants completed a questionnaire and permitted collection of nasal swab specimens from their show pigs. Before data were collected, multiple institutional review boards, the Institutional Animal Care and Use Committee of the University of Minnesota and the University of Iowa, and state fair officials approved the study.

We used the Centers for Disease Control and Prevention (CDC; Atlanta, GA, USA) real-time reverse transcription PCR (rRT-PCR) (*6*) to screen for influenza A virus. Swab specimens (run in duplicate) with cycle threshold (C_t_) values <35 were considered positive for influenza Avirus; specimens with C_t_ values of 35 to <40 were suspected to be positive; and specimens with C_t_ values >40 were considered negative. In a blinded fashion, aliquots of swab specimens from pigs were shared with the Minnesota Veterinary Diagnostic Laboratory (St. Paul, MN, USA), where rRT-PCRs for matrix, hemagglutinin (HA), and neuraminidase (NA) genes were performed. Specimens were then shared with the National Veterinary Services Laboratory (Ames, IA, USA) and later with CDC for further molecular and sequencing studies.

Positive and suspected-positive rRT-PCR specimens were cultured in shell vials on MDCK cells by using standard techniques. Sequence-based analyses of the influenza A virus isolates were performed by the CDC influenza division, using full or partial genome sequencing approaches for all 8 gene segments. Sequences were compared by using BLASTn alignment search techniques (http://blast.ncbi.nlm.nih.gov).

Questionnaires were completed by 121 (98%) participants. Participants were predominantly male (71%), and their median age was 34.9 years (range 9–75 years); 24% of participants were <18 years of age. Some pig exhibitors were children with <1 year of pig exposure (Table); others were pig farmers with numerous years of pig exposure. Participants reported an average of 18.7 years of pig exposure.

**Table:** Characteristics of 123 persons enrolled in study of influenza A virus among show pigs, Minnesota and South Dakota state fairs, 2008 and 2009

Characteristic	No. (%)
State fair where enrolled	
Minnesota	90 (73.2)
South Dakota	33 (26.8)
Year enrolled	
2008	48 (39.0)
2009	75 (61.0)
Age group, y*	
<8	29 (23.6)
18–39	37 (30.1)
40–59	46 (37.4)
>60	8 (6.5)
Sex*	
M	86 (69.9)
F	35 (28.5)
Work performed in the last 30 d†	
Unemployed	8 (6.5)
Farmer	108 (87.8)
Swine farmer	100 (81.3)
Government employee	8 (6.5)
Veterinarian/veterinary assistant	2 (1.6)
Receipt of vaccination for human influenza in past 2 y*	
Yes	35 (28.5)
No	76 (61.8)
Unknown	7 (5.7)
Respiratory illness in past 12 mo*	
Yes	64 (52.0)
No	55 (44.7)
Unknown	2 (1.6)
Worked with swine, y*	
<1	3 (2.4)
>1–4	10 (8.1)
5–10	39 (31.7)
>10	50 (40.7)

*These covariates have missing data. †Responses are not mutually exclusive.

Nasal swab specimens were collected from a total of 149 pigs (from Minnesota, 47 in 2008 and 57 in 2009; from South Dakota, 45 in 2009). Almost all (97%) swabbed pigs were <1 year of age, and 40% were female. All pigs were observed to be healthy by a veterinarian before they were permitted to enter the show.

In 2008, nasal swab specimens from show pigs showed no molecular or viral culture evidence of influenza A virus. However, in 2009, a number of pigs were positive for influenza A virus. Comparing the molecular results of the 3 laboratories and using conservative rRT-PCR result interpretations on which all laboratories agreed, we determined that influenza A virus was detected in 12 (12%) of 102 swine respiratory samples by rRT-PCR; 11 (19%) of these were from among the 57 pigs swabbed at the Minnesota state fair (Figure). Viral culture yielded 7 influenza isolates, 5 of which were recultured and sequenced by CDC. Sequence for 4 of the isolates (A/Swine/Minnesota/074A/2009, A/Swine/Minnesota/115A/2009, A/Swine/Minnesota/130A/2009, and A/Swine/Minnesota/136B/2009) were similar to influenza A(H1N1)pdm09 viral gene sequences: identity scores ranged from 99.8% to 100% for polymerase basic 1, polymerase basic 2, polymerase acidic protein, HA, nucleocapsid protein, NA, matrix, and nonstructural protein genes (Global Initiative on Sharing Avian Influenza Data [GISAID] accession nos. EPI295284–EPI295327). The fifth isolate (A/Swine/South Dakota/152B/2009) was cultured from an asymptomatic pig at the South Dakota state fair. After sequence studies of the HA and NA genes, this isolate was classified as a triple reassortant H1N2 virus (GISAID accession nos. EPI295328–EPI295335) similar to recent US swine isolates.

**Figure:**
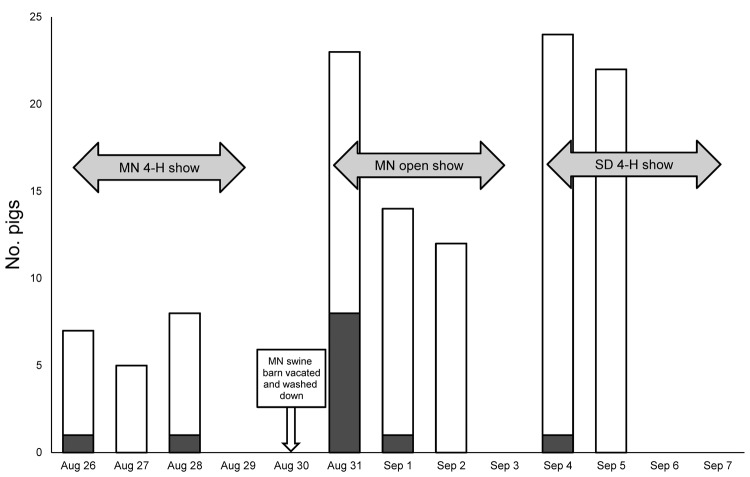
Show pigs with nasal swab specimens positive for influenza A virus by real-time reverse transcription PCR, Minnesota and South Dakota state fairs, 2009. MN, Minnesota; SD, South Dakota. White, total number of pigs swabbed; gray, pigs testing positive for influenza A by real-time reverse transcription PCR.

A follow-up telephone survey of study participants identified 2 with influenza-like illness (ILI) within 7 days after the fair: an adult, with ILI with onset 1 day after his pigs arrived at the fair (and 4 days before pig swabbing), and his daughter who developed an ILI on the last day of the fair (3 days after pig swabbing). Three pigs exhibited by the child tested positive for influenza A virus.

## Conclusions

We found a 19% prevalence of influenza A virus among the 57 show pigs swabbed at the 2009 Minnesota state fair, which occurred during the second wave of the 2009 pandemic. Temporal analysis of the results indicated that most pigs with rRT-PCR–positive results were sampled within 24 hours after arriving at the fair, suggesting that they probably were infected before their arrival (Figure). None of the pigs with molecular or viral culture evidence of influenza A virus infection had clinical signs of influenza illness at the time of sampling. This finding validates previous pig show–linked human cases (*5*,*7*) and suggests that exposure to apparently healthy pigs at shows is a possible source of influenza A virus transmission.

These detections of A(H1N1)pdm09 virus in the United States (reflecting the ease of transmission from humans to pigs) were soon followed by multiple other detections in US pigs (*8*,*9*) and in numerous pigs in other countries. Such observations are now common (*10*), leading to speculation that just as the human-origin subtype H3N2 virus variant became enzootic in pigs (*11*), the A(H1N1)pdm09 virus and related viruses are now enzootic. Our findings of asymptomatic A(H1N1)pdm09 virus infections in pigs is supported by other data suggesting that as few as 10% of infected pigs might show clinical signs of A(H1N1)pdm09 virus infection (*12*).

Of concern is that new reassortants between A(H1N1)pdm09, enzootic SIVs, and possibly other human- or avian-origin viruses might emerge and possibly spread to humans who have contact with asymptomatic pigs (*10*,*13*,*14*). Clinicians who care for persons in whom influenza A illness develops, particularly when human influenza is not widely circulating, should ask about pig exposures and consider further molecular testing to rule out human infection with a nonhuman-origin influenza A virus. In addition, to minimize potential interspecies transmission of influenza viruses, it might be prudent to develop guidelines for the exhibition of pigs.

Because of the possibility of novel virus generation in pigs and of human-to-pig and pig-to-human transmission of influenza virus, routine influenza A virus surveillance among pigs and influenza A virus testing of ill persons exposed to pigs is needed to ensure timely detection of novel influenza viruses in humans and pigs (*4*). Early detection is essential for development of effective vaccines and initiation of other means to prevent the spread of novel influenza A viruses. However, considerable barriers exist to conducting surveillance in pigs and pig-exposed persons, not the least of which is the threat that such surveillance could economically harm the pork industry (*10*). To improve influenza surveillance, additional ways are needed for pig farmers, the pork industry, the US Department of Agriculture, and public health professionals to collaborate. Swine workers, food animal veterinarians, and persons involved in raising show pigs are at high risk for zoonotic influenza infection (*4*,*15*). They should be strongly encouraged to receive seasonal influenza vaccines and to take measures to reduce zoonotic disease transmission, including using personal protective equipment. They also should limit their contact with pigs when they or the pigs have symptoms of respiratory illness (*4*,*10*).

## References

[1] Ma W, Lager KM, Vincent AL, Janke BH, Gramer MR, Richt JA. The role of swine in the generation of novel influenza viruses. Zoonoses Public Health. 2009;56:326–37. 10.1111/j.1863-2378.2008.01217.x 19486316

[2] Robinson JL, Lee BE, Patel J, Bastien N, Grimsrud K, Seal RF, Swine influenza (H3N2) infection in a child and possible community transmission, Canada. Emerg Infect Dis. 2007;13:1865–70. 10.3201/eid1312.070615 18258037 PMC2876760

[3] Olsen CW, Brammer L, Easterday BC, Arden N, Belay E, Baker I, Serologic evidence of H1 swine Influenza virus infection in swine farm residents and employees. Emerg Infect Dis. 2002;8:814–9. 10.3201/eid0808.010474 12141967 PMC2732505

[4] Gray GC, McCarthy T, Capuano AW, Setterquist SF, Olsen CW, Alavanja MC. Swine workers and swine influenza virus infections. Emerg Infect Dis. 2007;13:1871–8. 10.3201/eid1312.061323 18258038 PMC2876739

[5] Shinde V, Bridges CB, Uyeki TM, Shu B, Balish A, Xu X, Triple-reassortant swine influenza A (H1) in humans in the United States, 2005–2009. N Engl J Med. 2009;360:2616–25. 10.1056/NEJMoa0903812 19423871

[6] Centers for Disease Control and Prevention. CDC realtime RT-PCR. (rRTPCR) protocol for detection and characterization of influenza (version 2007). CDC ref. no. I-007–05. Atlanta: The Centers; 2007.

[7] Myers KP, Olsen CW, Gray GC. Cases of swine influenza in humans: a review of the literature. Clin Infect Dis. 2007;44:1084–8. 10.1086/512813 17366454 PMC1973337

[8] Yassine HM, Khatri M, Zhang YJ, Lee CW, Byrum BA, O'Quin J, Characterization of triple reassortant H1N1 influenza A viruses from swine in Ohio. Vet Microbiol. 2009;139:132–9. 10.1016/j.vetmic.2009.04.028 19477087

[9] Vincent AL, Swenson SL, Lager KM, Gauger PC, Loiacono C, Zhang Y. Characterization of an influenza A virus isolated from pigs during an outbreak of respiratory disease in swine and people during a county fair in the United States. Vet Microbiol. 2009;137:51–9. 10.1016/j.vetmic.2009.01.003 19203846

[10] Gray GC, Baker WS. Editorial commentary: the problem with pigs: it's not about bacon. Clin Infect Dis. 2011;52:19–22. 10.1093/cid/ciq051 21148515

[11] Vincent AL, Ma W, Lager KM, Janke BH, Richt JA. Swine influenza viruses a North American perspective. Adv Virus Res. 2008;72:127–54. 10.1016/S0065-3527(08)00403-X 19081490

[12] Forgie SE, Keenliside J, Wilkinson C, Webby R, Lu P, Sorensen O, Swine outbreak of pandemic influenza A virus on a Canadian research farm supports human-to-swine transmission. Clin Infect Dis. 2011;52:10–8. 10.1093/cid/ciq030 21148514 PMC3106227

[13] Kasowski EJ, Garten RJ, Bridges CB. Influenza pandemic epidemiologic and virologic diversity: reminding ourselves of the possibilities. Clin Infect Dis. 2011;52(Suppl 1):S44–9. 10.1093/cid/ciq010 21342899

[14] Liu Q, Ma J, Liu H, Qi W, Anderson J, Henry SC, Emergence of novel reassortant H3N2 swine influenza viruses with the 2009 pandemic H1N1 genes in the United States. Arch Virol. 2012;157:555–62. 10.1007/s00705-011-1203-9 22198410

[15] Myers KP, Olsen CW, Setterquist SF, Capuano AW, Donham KJ, Thacker EL, Are swine workers in the United States at increased risk of infection with zoonotic influenza virus? Clin Infect Dis. 2006;42:14–20. 10.1086/498977 16323086 PMC1673212

